# Structural Transformations in Austenitic Stainless Steel Induced by Deuterium Implantation: Irradiation at 295 K

**DOI:** 10.1186/s11671-016-1251-x

**Published:** 2016-02-01

**Authors:** Oleksandr Morozov, Volodymir Zhurba, Ivan Neklyudov, Oleksandr Mats, Viktoria Progolaieva, Valerian Boshko

**Affiliations:** National Science Center “Kharkiv Institute of Physics and Technology”, 1, Akademichna St., UA-61108 Kharkiv, Ukraine

**Keywords:** Structural transformations, Austenitic stainless steel, Deuterium, Ion implantation, Irradiation at 295 K, TDS, TEM, 81.40.Wx, 81.70.Pg, 81.40.-z

## Abstract

Deuterium thermal desorption spectra were investigated on the samples of austenitic steel 18Cr10NiTi pre-implanted at 295 K with deuterium ions in the dose range from 8 × 10^14^ to 2.7 × 10^18^ D/cm^2^. The kinetics of structural transformation development in the steel layer was traced from deuterium thermodesorption spectra as a function of deuterium concentration. Three characteristic regions with different low rates of deuterium amount desorption as the implantation dose increases were revealed: I—the linear region of low implantation doses (up to 1 × 10^17^ D/cm^2^); II—the nonlinear region of medium implantation doses (1 × 10^17^ to 8 × 10^17^ D/cm^2^); III—the linear region of high implantation doses (8 × 10^17^ to 2.7 × 10^18^ D/cm^2^). During the process of deuterium ion irradiation, the coefficient of deuterium retention in steel varies in discrete steps. Each of the discrete regions of deuterium retention coefficient variation corresponds to different implanted-matter states formed during deuterium ion implantation. The low-dose region is characterized by formation of deuterium-vacancy complexes and solid-solution phase state of deuterium in the steel. The total concentration of the accumulated deuterium in this region varies between 2.5 and 3 at.%. The medium-dose region is characterized by the radiation-induced action on the steel in the presence of deuterium with the resulting formation of the energy-stable nanosized crystalline structure of steel, having a developed network of intercrystalline boundaries. The basis for this developed network of intercrystalline boundaries is provided by the amorphous state, which manifests itself in the thermodesorption spectra as a widely temperature-scale extended region of deuterium desorption (structure formation with a varying activation energy). The total concentration of the accumulated deuterium in the region of medium implantation doses makes 7 to 8 at.%. The resulting structure shows stability against the action of deuterium ion implantation. This manifests itself in a nearly complete ceasing of deuterium accumulation from a newly implanted dose (radiation-resistant structure).

## Background

The investigation into regularities of hydrogen interaction with metals and alloys over a wide range of temperatures and pressures still remains a currently central problem in material physics from both the scientific and practical standpoints [1–9]. The hydrogen accumulation in structural and functional materials is an extremely hazardous phenomenon, which leads to hydrogen degradation of materials and to possible unforeseen equipment failures [10–14]. The degradation of materials increases due to hydrogen interaction with the whole range of crystal structure imperfections of solids such as interstitial and substitutional impurities, vacancies and their complexes, dislocations and their pileups, subgrain and grain boundaries, and phase components [11, 15, 16].

Vehoff [17] has reviewed the interaction of hydrogen with defects in metals. He states that hydrogen concentrations far in excess of the lattice concentration cannot be produced by elastic interactions alone, because the amount of hydrogen in the so-called Cottrell atmosphere around a dislocation is small. In contrast, chemical interaction or trapping at preferred lattice sites such as grain boundaries, particle-matrix interfaces, or dislocation cores can lead to a rise in the local hydrogen concentration that can span many orders of magnitude. The respective concentration increase is highly dependent on the local chemical and/or structural arrangement [18–23].

Temperature, mechanical, radiation, and implantation effects give rise to additional structural changes in metals, alloys, and steels. These are the induced phase transformations, an increase in the density of dislocations and vacancies and pair production. All these factors enhance the hydrogen accumulation and lead to the loss of plasticity, and later on, to the failure [11, 12, 24–29].

Hydrogenation of the face-centered cubic (fcc) iron-based alloys, which constitute an array of austenitic stainless steels, can cause the following phase transformations: fcc (***γ***) → bcc (α*) and fcc (***γ***) → hcp (***ε***) [12, 30–35]. The hydrogen-induced phases are sometimes considered as pseudohydrides. It is generally assumed that the role of hydrogen consists in the creation of a particular stress state that triggers the phase transformation. In contrast, Vakhney and co-workers [36] showed that the phase transformation fcc (***γ***) → hcp (***ε***) was also due to a change in the electronic structure of the alloy after hydrogenation. At the same time, several studies have shown that martensite formation is responsible for hydrogen-enhanced crack growth, which results in quasi-cleavage fracture of specimens tested in hydrogen [37, 38]. Gey and co-workers [39] found that the amount of strain-induced ***ε*-** and α*- martensite in AISI 304 steel (Pittsburgh, PA, USA) was strongly dependent on the local mutual orientations of neighboring grains, i.e., texture of the ***γ*** steel.

Stainless steel is one of the most useful classes of engineering materials. For example, austenitic steel is used for manufacturing vessel internals of fission-type reactors. A wide use of austenitic stainless steels as structural fission reactor elements calls for a detailed knowledge of their behavior under conditions of radiation influence, accumulation of gas impurities (hydrogen isotopes, above all) [12–14, 19, 26–29, 40, 41].

At saturation of austenitic stainless steel with deuterium at 100 K by means of ion implantation, structural-phase changes take place, depending on the dose of implanted deuterium. The increase in the implanted dose of deuterium is accompanied by the increase in the retained deuterium content, and as soon as the deuterium concentration reaches C ≈ 0.5, the process of shear martensitic structural transformation in steel takes place. It includes the formation of characteristic bands, body-centered cubic (bcc) crystal structure, and ferromagnetic phase. On reaching the concentration C ≥ 0.5, two hydride phases are formed in the steel, the decay temperatures of which are 240 and 275 K. The maximum attainable concentration of deuterium in steel is C = 1 (at.D/at.met. = 1/1) by irradiation at 100 K [42].

One of the most informative methods of investigating hydrogen behavior in metals is the thermal desorption spectrometry (TDS). The TDS technique enables one to determine the temperature ranges of implanted hydrogen retention and release, to find thermoactivation parameters, and to establish quantitative characteristics of hydrogen emission-reemission. It also shows fair correlation between the thermoactivated hydrogen release spectra and the metal-hydrogen phase diagrams [43–45].

The present paper reports the results from studies on implanted deuterium-induced structural transformations in austenitic stainless steel 18Cr10NiTi as functions of implanted deuterium doses at a temperature of 295 K. The structural transformation kinetics in the implanted steel layer has been traced from the deuterium thermodesorption (TD) spectra versus implanted deuterium concentrations. The important methodical advantage of the present study lies in the fact that the implantation of the assigned dose of deuterium into steel and the measurements of temperature ranges of deuterium desorption were performed in one and the same setup (in situ).

## Methods

The TDS technique has been used to investigate the kinetics of spectrum development for deuterium desorption from austenitic stainless steel 18Cr10NiTi versus the implanted deuterium dose. The chamber is partitioned into two separate, nearly equal in volume compartments: analytical and pumping. At the center of the partition wall, there is an iris diaphragm with the aperture variable in the range from 10 to 50 mm. That permits one to control the rate of pumping of the analytical compartment and to increase, if necessary, the sensitivity of the analyzer registering the partial gas pressure in the chamber. The opening of the diaphragm is controlled from the outside of the chamber by means of the magnetic system. In our TDS measurements, we have used the Type APPM-1 monopolar mass-spectrometer. In our experiments, to reduce the impact of background hydrogen being present in the samples and in the target chamber, we have used deuterium as the hydrogen isotope. The sample irradiation and the TDS measurements were carried out using the experimental facility “SKIF” described in detail in ref. [46].

The samples were pre-implanted with 12 keV deuterium ions (${D}_2^{+}$, 24 keV) at current density 5 μA/cm^2^ in the dose range from 8 × 10^14^ to 2.7 × 10^18^ D/cm^2^ at the sample temperature *T*_irr._ = 295 K. The mass separator provides the resolution of ion beams with ion masses ranging between 40 and 50 amu, m/Δm ~ 50.

The austenitic stainless steel 18Cr10NiTi samples (Table 1), measuring 10 × 5 × 0.3 mm^3^, were fastened to the same-steel heaters, measuring 40 × 5 × 0.3 mm^3^. The radiation area was *S* = 0.3 cm^2^. Prior to irradiation, the samples underwent short-time annealing (1 min.) at 1350 K in order to degas them and to remove impurities from their surface. After irradiation, the samples implanted to the pre-assigned dose were heated in the same measurement chamber at a rate of ~3.5 K/s to a temperature of ~1700 K, with simultaneous registration of the ${D}_2^{+}$ ion desorption spectrum (4 amu). The heating of samples was turned on immediately after the ion beam was switched off. The temperature was measured by the tungsten-rhenium thermocouple WRe 5/20 fixed to the sample. The total amount of deuterium released from the sample was determined from the area lying under the gas-release curve.

**Table 1:** Chemical composition of austenitic stainless steel 18Cr10NiTi (weight percent)

С	Cr	Ni	Ti	Mn	Si	P	S	Fe
Around 0.1	17.1 to 18.5	9.6 to 10.2	0.45 to 0.56	1.0 to 1.2	0.4 to 0.43	0.03	0.01	Rest

The sample structure was examined by means of transmission electron microscopy at an accelerating voltage of 100 kV. The steel samples were thinned down to a thickness of 50 to 60 μm through chemical etch polishing, following which deuterium ions were implanted to the prescribed dose. Thereafter, disks, 3 mm in diameter, were cut out from the obtained foil (the diameter was specified by the sample holder for the microscope), and a final thinning was done in the same solution.

The combination of two techniques, viz., thermal desorption mass-spectrometry and ion implantation, makes it possible (through variation of implantation doses in a wide range and with different steps) to establish the interrelation between the quantity of implanted deuterium, the retention coefficient, and the kind of desorption spectrum.

## Results and Discussion

### The Typical Deuterium Thermodesorption Spectra

**Fig. 1:**
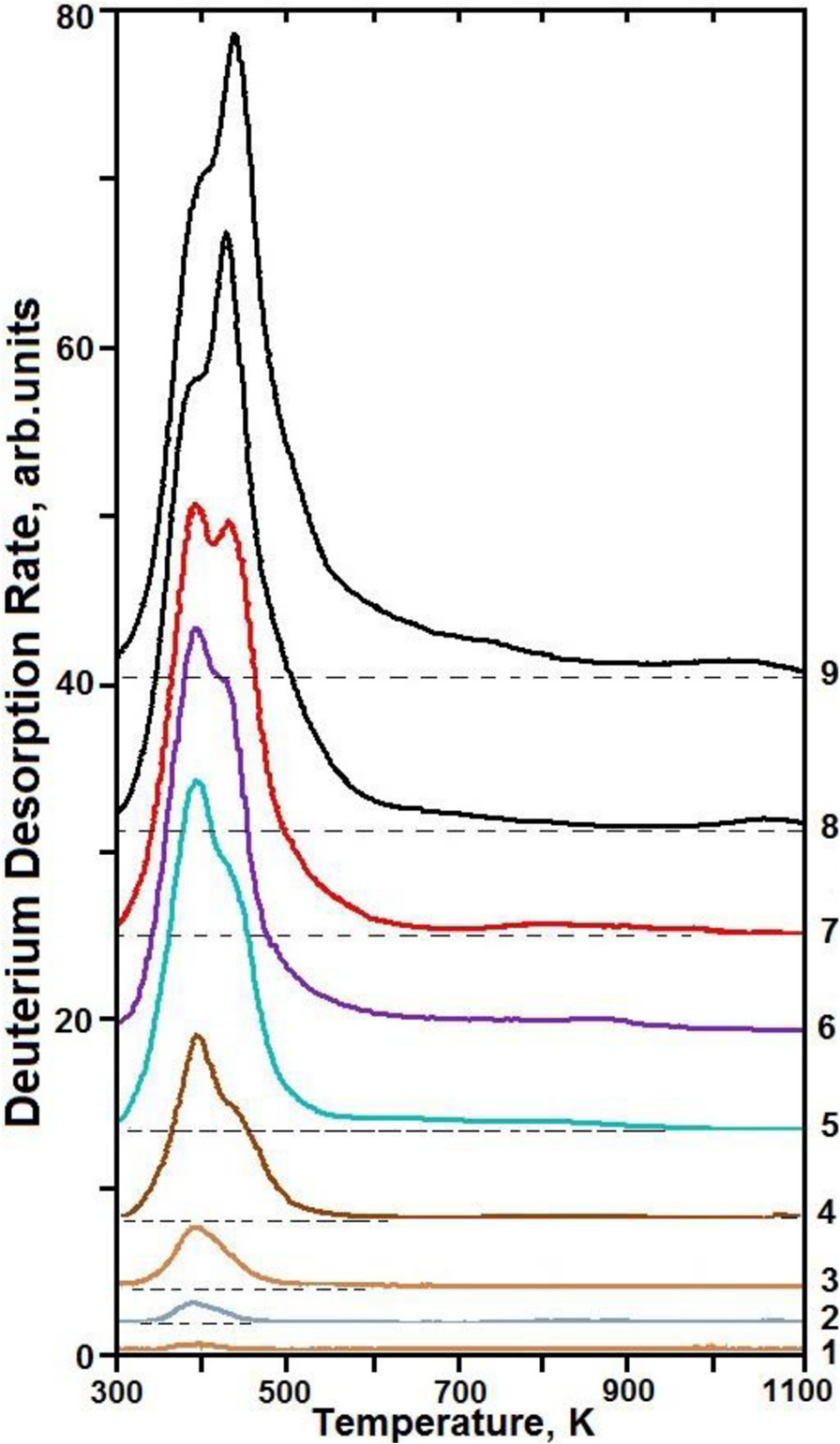
Deuterium TD spectra from austenitic steel samples implanted with different doses of deuterium ions: (*1*)—3.5 × 10^15^ D/сm^2^; (*2*)—1.1 × 10^16^ D/сm^2^; (3)—3.3 × 10^16^ D/сm^2^; (*4*)—1.0 × 10^17^ D/сm^2^; (*5*)—3.5 × 10^17^ D/сm^2^; (*6*)—5.5 × 10^17^ D/сm^2^; (*7*)—8.0 × 10^17^ D/сm^2^; (*8*)—2.2 × 10^18^ D/сm^2^; (*9*)—2.7 × 10^18^ D/сm^2^

The typical deuterium TD spectra measured at different doses of implanted deuterium ions are presented in Fig. 1. It can be seen that at low implantation doses, the TD spectrum shows no explicit gas-release peaks and represents a temperature-wide region of desorption. With an increasing deuterium implantation dose in this range of temperatures, the TD spectrum shows the peaks, the intensity of which is dependent on the dose of implanted deuterium. A correlation has been found between different implanted substance states, formed during the process of deuterium ion implantation, and the TD spectrum structure. The kinetics of the deuterium TD spectrum behavior has been analyzed in detail as a function of the deuterium implantation dose. The analytical results will be presented below.

### Discrete Character of Variation in the Amount of Retained Deuterium with an Increasing Implantation Dose

**Fig. 2:**
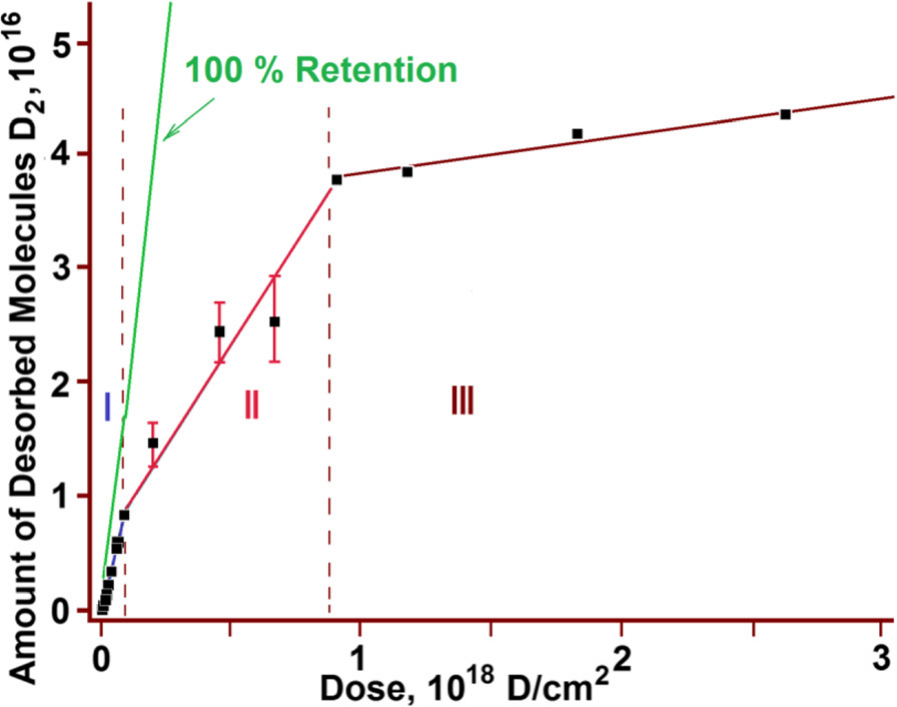
Amount of desorbed deuterium versus irradiation dose for austenitic steel implanted at 295 K

The studies carried out in the implantation dose range from 8 × 10^14^ to 2.7 × 10^18^ D/cm^2^ have revealed a nonlinear growth in the amount of released deuterium *Q*(*F*) as the radiation dose (*F*) increased (see Fig. 2). It is seen from the figure that the function *Q*(*F*) does not attain a plateau, this pointing to the fact that at room temperature, in the implantation dose range under study, the full saturation of the sample with deuterium is not attained.

In the plot, three characteristic regions with different Q(F) patterns can be distinguished:

- I—the linear region of low implantation doses (up to 1 × 10^17^ D/cm^2^);
- II—the nonlinear region of medium implantation doses (1 × 10^17^ to 8 × 10^17^ D/cm^2^);
- III—the linear region of high implantation doses (8 × 10^17^ to 2.7 × 10^18^ D/cm^2^).

For a further constructive discussion, it stands to mention an important peculiarity of the obtained experimental results. In regions I and III, the function *Q*(*F*) has a linear character with insignificant scatter of the experimental points, thereby testifying to sufficiently advanced methods employed for TDS studies. In turn, this permits us to state that the scatter of the experimental points observed in region II is not caused by procedural errors in the performance of the experiment.

Different slope angles of straight-line segments in the *Q*(*F*) plot reflect the discrete character of variation in the amount of retained deuterium with an increasing implantation dose. The stability of radiation characteristics (target temperature, implanted ion energy, and current density) in the process of experiments allows the conclusion that *each of the discrete implantation dose corresponds to a different state of the implanted matter formed during deuterium ion implantation*.

It is quite reasonable to assume that in region I that reflects the invariable rate of deuterium accumulation up to a dose of 1 × 10^17^ D/cm^2^, the matter is in the initial state from the viewpoint of deuterium retention kinetics. This is witnessed by the unvarying law of linearly increasing amount of implanted deuterium with an increase in the implantation dose. Then, accordingly, the linear region III may also characterize the stable state of the implanted matter having a lower coefficient of deuterium retention.

In the given context, a wide scatter of the experimental points in region II can be attributed to the increasing probabilistic nature of deuterium retention in the implanted dose range 1 × 10^17^ to 8 × 10^17^ D/cm^2^. This enables us to define the state of implanted matter as transient between stable states I and III.

**Fig. 3:**
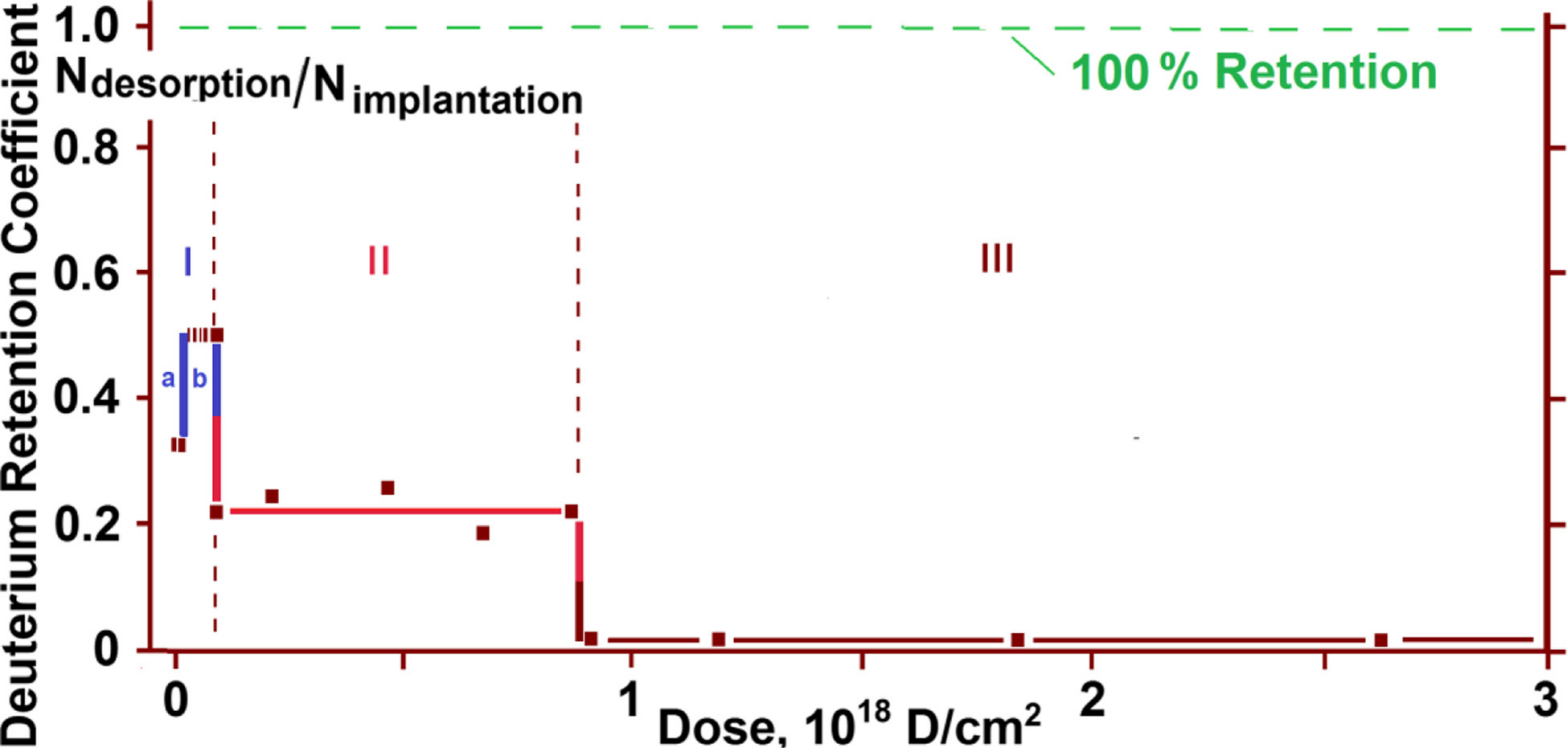
Deuterium retention coefficient as a function of the implantation dose

For each experimental point, deuterium retention coefficients were calculated; the obtained values were plotted against the implantation dose (Fig. 3). The amount of retained deuterium was estimated with due account for the number of implanted deuterium atoms and the number of desorbed deuterium molecules.

In region I, the deuterium retention coefficient is the highest. Here, note that at low implanted deuterium doses (area I***a***), it equals ~35 %, while with a dose increase over 1 × 10^16^ D/cm^2^, it increases up to ~50 % (area I***b***). The second region (II) is characterized by a decrease in the deuterium retention coefficient down to ~23 %. In region III, the deuterium retention coefficient is sharply and considerably reduced down to ~2 %. A slow accumulation of implanted deuterium against the background of a high level of reemission in region III is observed up to a dose of 2.7 × 10^18^ D/cm^2^, which was ultimate in our experiments.

Undoubtedly, the obtained pattern of deuterium retention coefficient values qualitatively reflects the *stepwise character* of the process of deuterium ion implantation in austenitic steel samples as the implantation dose increases.

The linear regions I and III characterize a stable process of deuterium implantation with a constant retention coefficient in the corresponding dose range. Region I corresponds to the metal state with a low-dose ion implantation, and consequently, with a low level of radiation action, being close in its structure to the initial state. Since the radiation conditions (kind and energy of implanted ions, radiation temperature and current density) were kept the same during implantation, the variation in the retention coefficient values as the dose of implanted deuterium exceeds 1 × 10^17^ D/cm^2^ may point to the increase of reemission and reflection characteristics of the steel samples themselves. This, in its turn, may be caused by some change in the nature of the implanted material, or more exactly, by structural rearrangements in the depth profile of deuterium, which occur due to the action of radiation and the increasing concentration of implanted deuterium.

Unlike region I, region III reflects the process of deuterium implantation into the material with a high degree of radiation action, i.e., with a high degree of structure imperfection. The foregoing suggests the conclusion that owing to deuterium irradiation, the metal goes over from one stable structural state with a constant trapping coefficient to the other radiation-resistant structural state, also characterized by a constant trapping coefficient.

A significant scatter of deuterium trapping coefficient values in region II may testify that the process of stable structure state formation takes place, during which the coefficient of deuterium retention is unstable.

For sure, when determining the nature and kinetics of implanted hydrogen capture and retention in austenitic steels, it is necessary to consider the integrated effect of the two aspects of the implantation process: (i) the growth of implanted hydrogen atom concentration and (ii) the increased defect formation as a result of the radiation effect. The hydrogen concentration increase can result in the formation of ordered hydrogenous structures, whereas the radiation-induced point defects and their complexes can give rise to additional traps for hydrogen capture and retention. The synergetics of these multilevel actions can lead to modified states of the initial material. Therefore, of apparent interest is the investigation into the evolution of the TD spectrum with an increasing implantation dose of hydrogen (in our case, deuterium).

### Evolution of Deuterium TD Spectrum with an Increasing Implantation Dose

Keeping in mind the stepwise pattern of deuterium retention in austenitic steel, we now consider the evolution of the deuterium thermodesorption spectrum structure with an increasing implantation dose and try to find out the regularities for each of the three stages of deuterium implantation and retention.

**Fig. 4:**
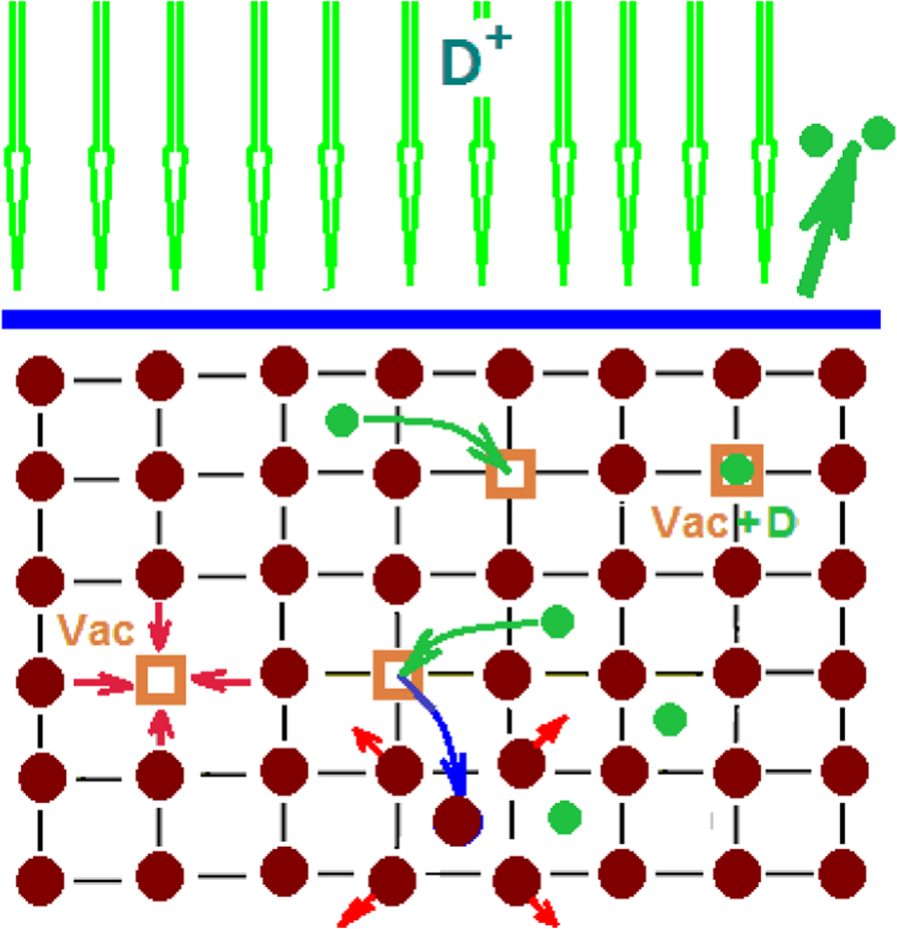
Processes occurring as a result of ion implantation: *dark red circle*—matrix atoms; *green circle*—interstitial atoms; —vacancy; —back-scattered atoms; —vacancy-deuterium complex

It should be noted that the process of deuterium implantation proceeds against the background of accompanying processes, among which we should mention the back scattering of incident ions and reemission of atoms as the target gets saturated (see Fig. 4). Depending of the incident particle energy, a different number of matrix structure damages are formed, and first of all, we mention Frenkel defects, viz., interstitial atoms and vacancies. The SRIM code [47] computations have shown that each implanted ion of mass 2, 12 keV energy gives impetus to formation of 27–28 vacancies. At the same time, according to the literature data, practically all Frenkel pairs formed during irradiation recombine at room temperature [48].

In the present experiments, the *important peculiarity of sample irradiation consists in* the presence of the contribution from the radiation component that accompanies the process of deuterium ion implantation.

Having a high diffusion mobility, deuterium atoms can exert an essential effect on the inhibition of Frenkel defect recombination through formation of vacancy-hydrogen complexes. For example, the data calculated by B.L. Shivachev [49] indicate the increase in the lifetime of defects in hydrogen-containing nickel.

#### Low Deuterium Concentrations—Region I

**Fig. 5:**
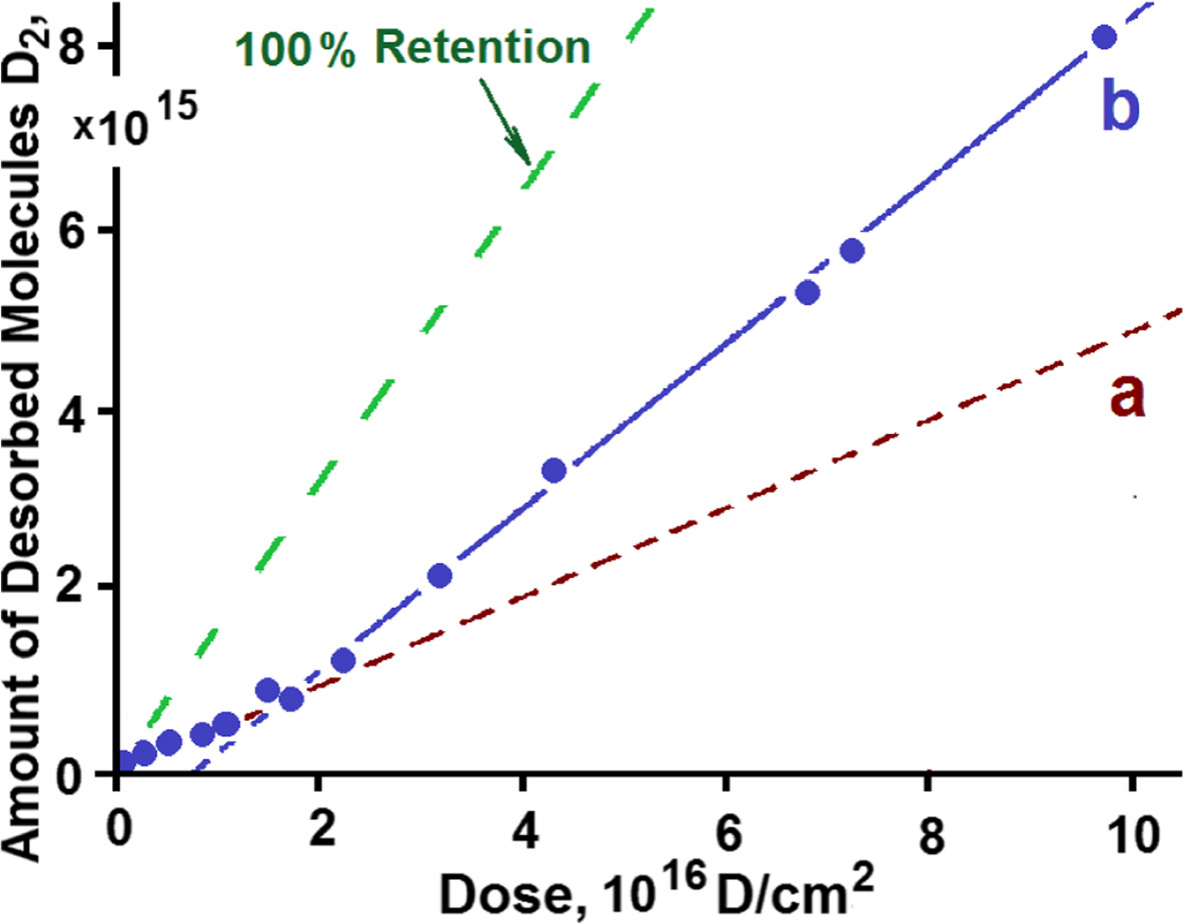
Amount of deuterium *Q*(*F*) desorbed from austenitic steel samples exposed to low doses of deuterium ions

A close consideration of the integrated saturation curve (region I) for austenitic steel samples (see Fig. 2) has revealed its kink in the implantation dose range 1.0 × 10^16^ D/cm^2^ (see Fig. 5).

A sufficient number of the experimental points and the order of their position in the plot permit us to represent region I as two successive linear segments ***a*** and ***b***, which are characterized by different slope angles with respect to the abscissa axis (dose axis). This, in turn, gives evidence of changes in the hydrogen trapping kinetics during irradiation (see Figs. 3 and 5).

**Fig. 6:**
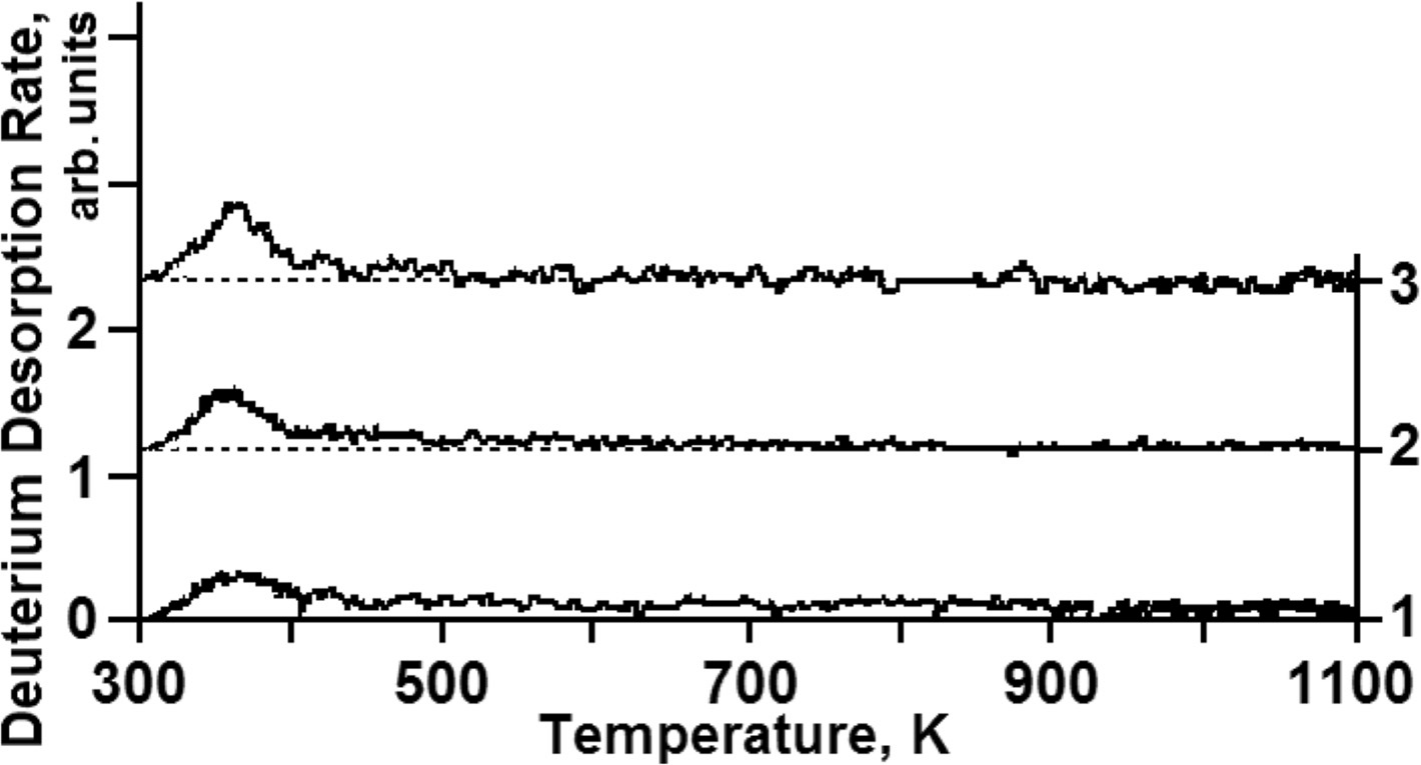
TD spectra of deuterium implanted in austenitic steel at radiation doses: (*1*)—8 × 10^14^ D/cm^2^; (*2*)—1.6 × 10^15^ D/cm^2^; (*3*)—2.5 × 10^15^ D/cm^2^

The spectrum of deuterium thermodesorption from the samples exposed to doses below 1 × 10^16^ D/cm^2^ (corresponding to segment ***a*** in Figs. 3 and 5) represents the temperature-scale smeared region of deuterium desorption with undetectable maxima in the temperature range between 330 and 400 K (see Fig. 6). It is reasonable to assume that at low radiation doses, deuterium is retained in the vicinity of randomly distributed structural formations, vacancies already in existence and formed during irradiation. In the process, deuterium-vacancy complexes are also formed.

**Fig. 7:**
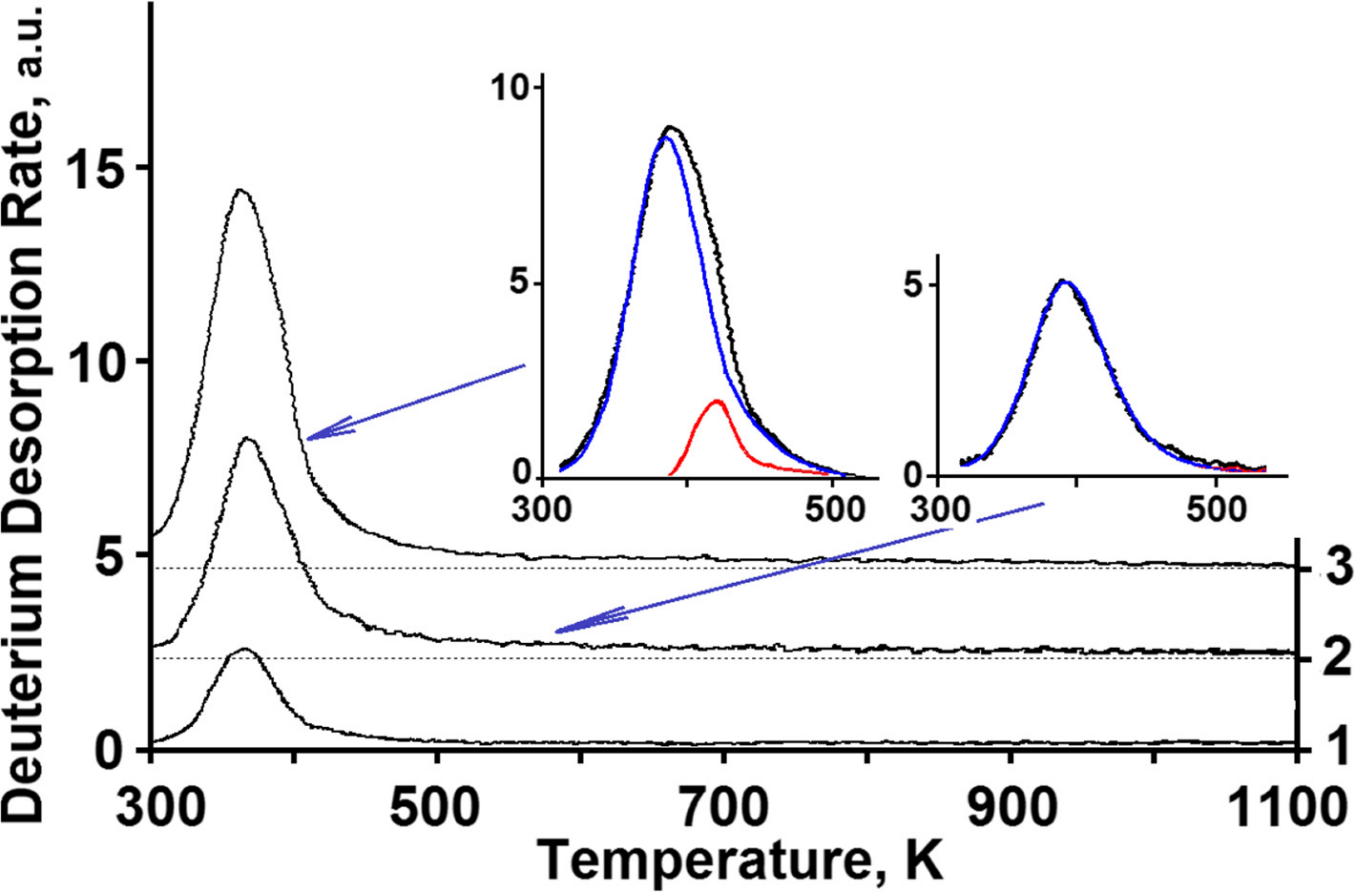
TD spectra of deuterium implanted in austenitic steel at radiation doses: (*1*)—3.5 × 10^15^ D/cm^2^; (*2*)—1.1 × 10^16^ D/cm^2^; (*3*)—3.3 × 10^16^ D/cm^2^

**Fig. 8:**
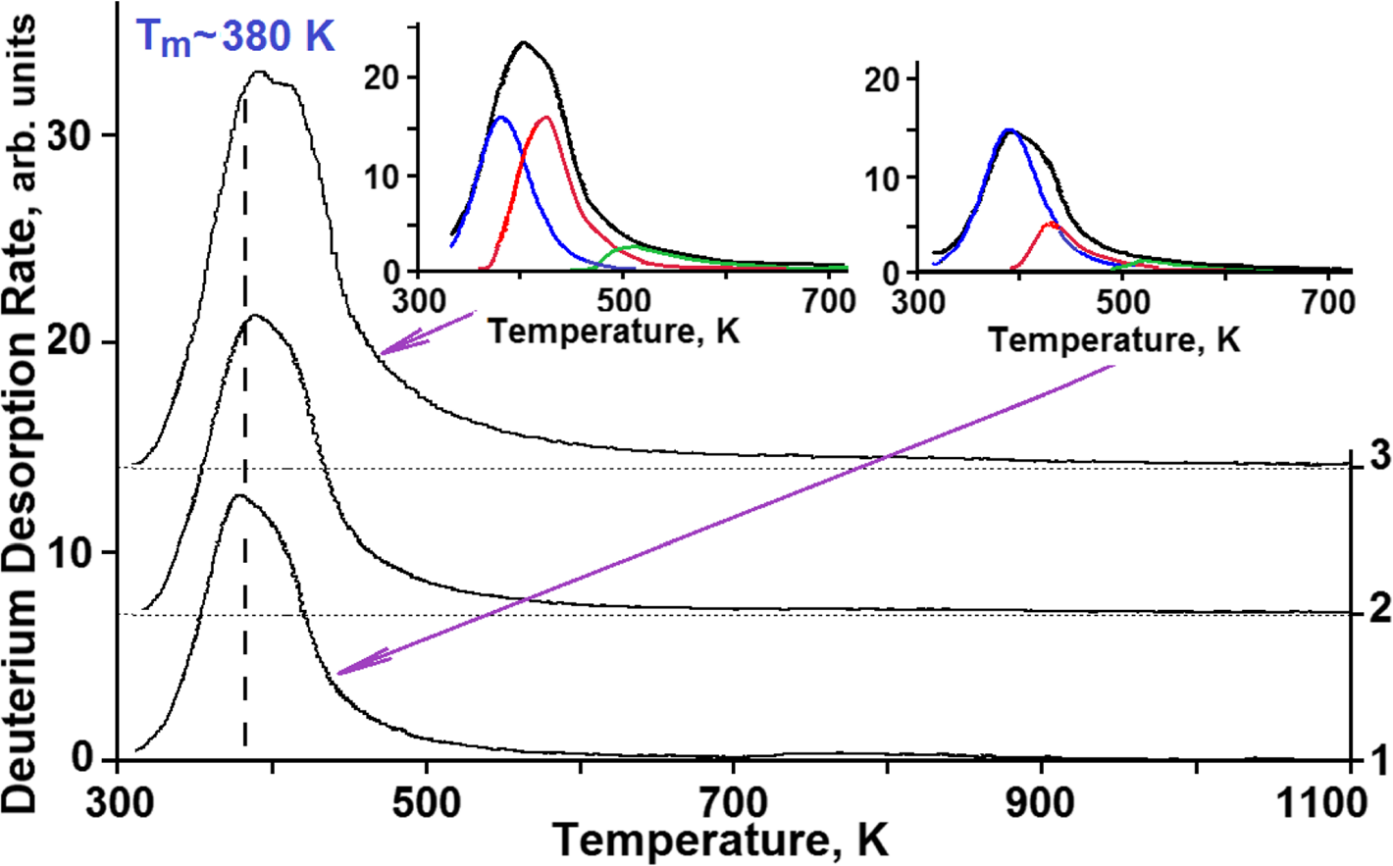
TD spectra of deuterium implanted in austenitic steel at radiation doses: (*1*)—1 × 10^17^ D/cm^2^; (*2*)—3.5 × 10^17^ D/cm^2^; (*3*)—5.5 × 10^17^ D/cm^2^

An increase in the implantation dose in excess of 1 × 10^16^ D/cm^2^ (see Fig. 5, segment ***b***) leads to a substantial increase in the deuterium retention coefficient (Figs. 3 and 5) from ~35 % up to ~50 %, and thus, to reduced reemission, as well as to manifestation of a clearly marked region of deuterium desorption with the peak temperature of 380 K (see Fig. 7). The intensity of the peak increases up to a dose of 1 × 10^17^ D/cm^2^ (see Fig. 8). The presence of a single peak with an invariable maximum temperature (*T*_m_) in the TD low-dose spectral region gives grounds to suggest that it is determined by the formation of a *solid solution of deuterium in steel* (ordered deuterium atom arrangement in steel).

As the estimations show, the formation of deuterium solid solution in steel starts as the deuterium integral concentration in the implantation layer reaches ~1 at.% and comes to an end as the deuterium concentration ~2.5 at.% is reached. The completion of the state formation is evidenced by the kink of the curve that shows the amount of desorbed deuterium versus the radiation dose for steel (see Fig. 2), and also, by the peak intensity growth termination with the *T*_m_ = 380 K in the deuterium TD spectrum (see Fig. 8, the insets with the peak partition into constituents—curves 1 and 3). Note that in this case, we also observe the appearance and intensity growth of an additional high-temperature region of deuterium desorption with the *T*_m_ at ~440 K. This region is observed in the deuterium TD spectrum as an expansion of the high-temperature region of the spectrum.

**Fig. 9:**
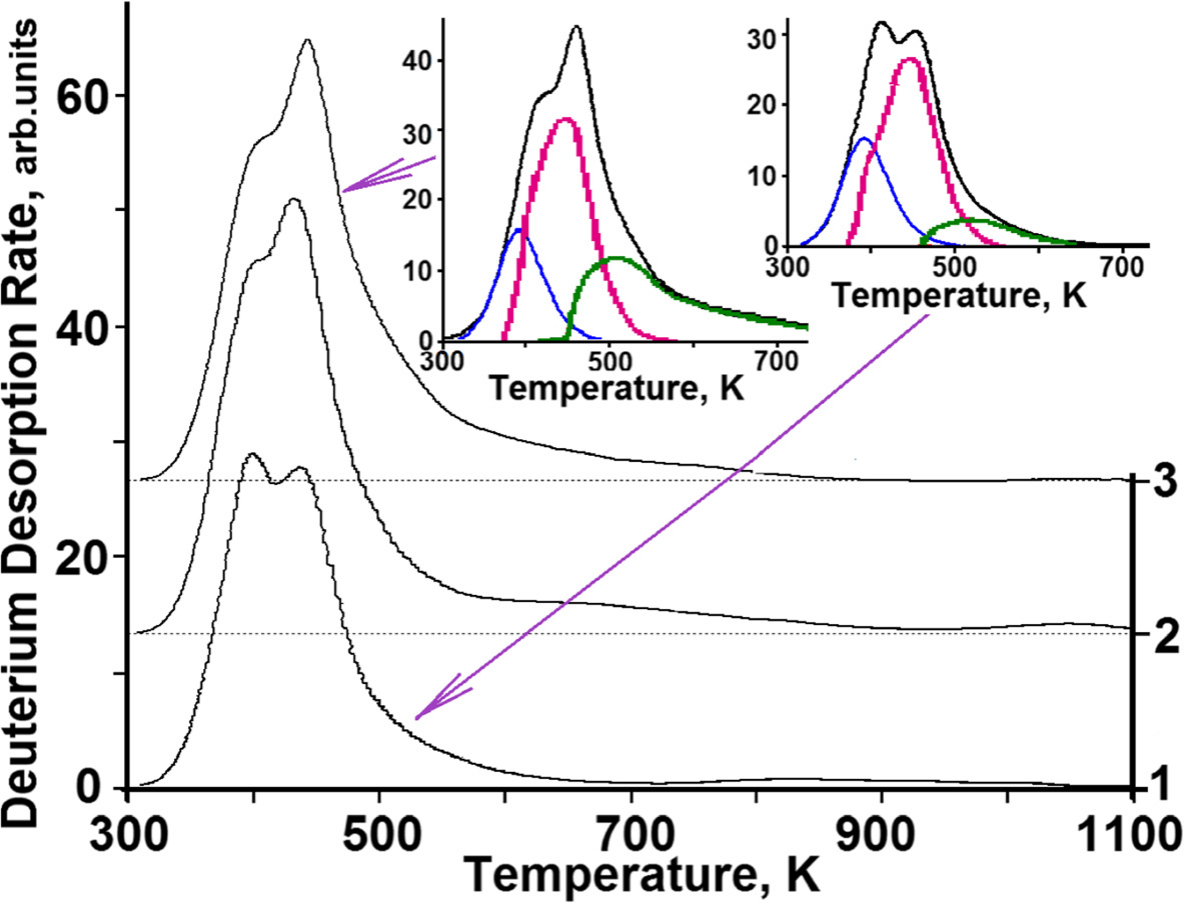
TD spectra of deuterium implanted in steel at radiation doses: (*1*)—8.0 × 10^17^ D/cm^2^; (*2*)—2.2 × 10^18^ D/cm^2^; (*3*)—2.7 × 10^18^ D/cm^2^

Figures 7, 8, and 9 show the peak partition curves calculated with the use of Arrhenius equations.

#### The Region of Medium Implantation Doses—Region II

Region II is characterized by a decrease in the deuterium retention coefficient, and also, by the greatest increase in the amount of retained deuterium (see Fig. 2). The TD spectrum of deuterium shows up two regions of deuterium desorption with *T*_m_ temperatures of ~380 and ~440 K (see Fig. 8). The amount of retained deuterium increases with the increasing implantation dose, owing to the intensity growth of the high-temperature spectral region. In this case, the amount of retained deuterium in the low-temperature part of the spectrum (the peak with *T*_m_ ~ 380 K) remains practically unchanged. This process lasts up to a dose of 8 × 10^17^ D/cm^2^.

The total concentration of accumulated deuterium in the medium-dose region forms the structure, which retains 7 to 8 at.% of deuterium.

Here, as is evident from Fig. 2, we observe the widest scatter of the experimental points. A considerable scatter of deuterium trapping coefficient values in region II may indicate that the process of stable structural state formation has a probabilistic nature. This implies that on completion of the deuterium solid-solution formation in steel, the newly implanted deuterium atoms promote further radiation-induced structural transformations.

The electron-microscope studies reveal the formation of a great number of small precipitates, which present ordered regions in some places (Fig. 11b).

#### The Region of High Implantation Doses—region III

At doses in excess of 8 × 10^17^ D/cm^2^, the mechanism of implanted deuterium retention changes. This manifests itself in a sharp fall of the deuterium retention coefficient down to 2 %, and also, in the kink of the curve showing this coefficient as a function of the radiation dose (see Figs. 2 and 3). The obtained data indicate that the prolonged deuterium ion implantation has caused structural transformations in the steel samples with the result that nearly all newly implanted deuterium gets desorbed from the steel. These data encourage us to draw a conclusion about the formation of a new structure, which is resistant to the action of deuterium ion implantation (*radiation*-*resistant structure*).

The parts of deuterium TD spectra, which correspond to region III of implantation doses, show three temperature ranges of deuterium desorption:

- The peak with the *T*_m_ = 380 K (solid solution of deuterium in steel) with retained deuterium concentration ranging from 2.5 to 3 at. %;
- The peak with the *T*_m_ = 440 K with the retained deuterium concentration of 7 to 8 at.%;
- Low-intensity, temperature-scale-wide region of deuterium desorption with a poorly marked temperature peak in the range of about 500 K.

The main contribution to the increase in the retained deuterium intensity in the high-dose region (region III) is provided by the increase in the intensity of the temperature-scale-wide region of deuterium desorption in the temperature range from 450 to 900 K (see Fig. 9) at a rate of ~2 % of the implantation dose. In this case, the quantity of desorbed deuterium in other temperature regions remains practically unchanged. Note that the low-intensity, temperature-scale-wide region of deuterium desorption became clearly seen in the deuterium TD spectrum at doses exceeding 5 × 10^17^ D/cm^2^ (see Fig. 8, curve 3).

The observed slow buildup of retained deuterium in steel (see Fig. 2) is evidently caused by implanted deuterium diffusion beyond the implantation volume, which is specified by both the increased deuterium concentration in the implantation volume (concentration gradient) and the formation of the transition deuterium-filled layer between the implanted and original areas of the sample.

A high concentration of ordered formations with a developed network of intercrystalline boundaries is characteristic of region III (see Fig. 11c).

The low-intensity, temperature-scale-wide region of deuterium desorption, observed in the TDS spectrum, can testify to the formation of the amorphous phase in steel, which evidently lies at the basis of intercrystalline boundaries formed in the steel subjected to long-term high-dose irradiation with deuterium ions.

The long-term irradiation of steel with deuterium ions results in the formation of the structure based on nanosized crystallites with an extensive network of intercrystalline boundaries. The basis for this multibranch network is the amorphous state, which is manifested in the TDS spectra as a temperature-scale-wide region of deuterium desorption (structural formation with variable activation energy). The structure resulting from deuterium ion implantation shows stability against the action of deuterium ion implantation. This makes itself evident in a nearly entire cessation of deuterium accumulation from newly implanted ions (*radiation*-*resistant structure*).

### Amount of Deuterium in Each Individual Peak of the Thermodesorption Spectrum

**Fig. 10:**
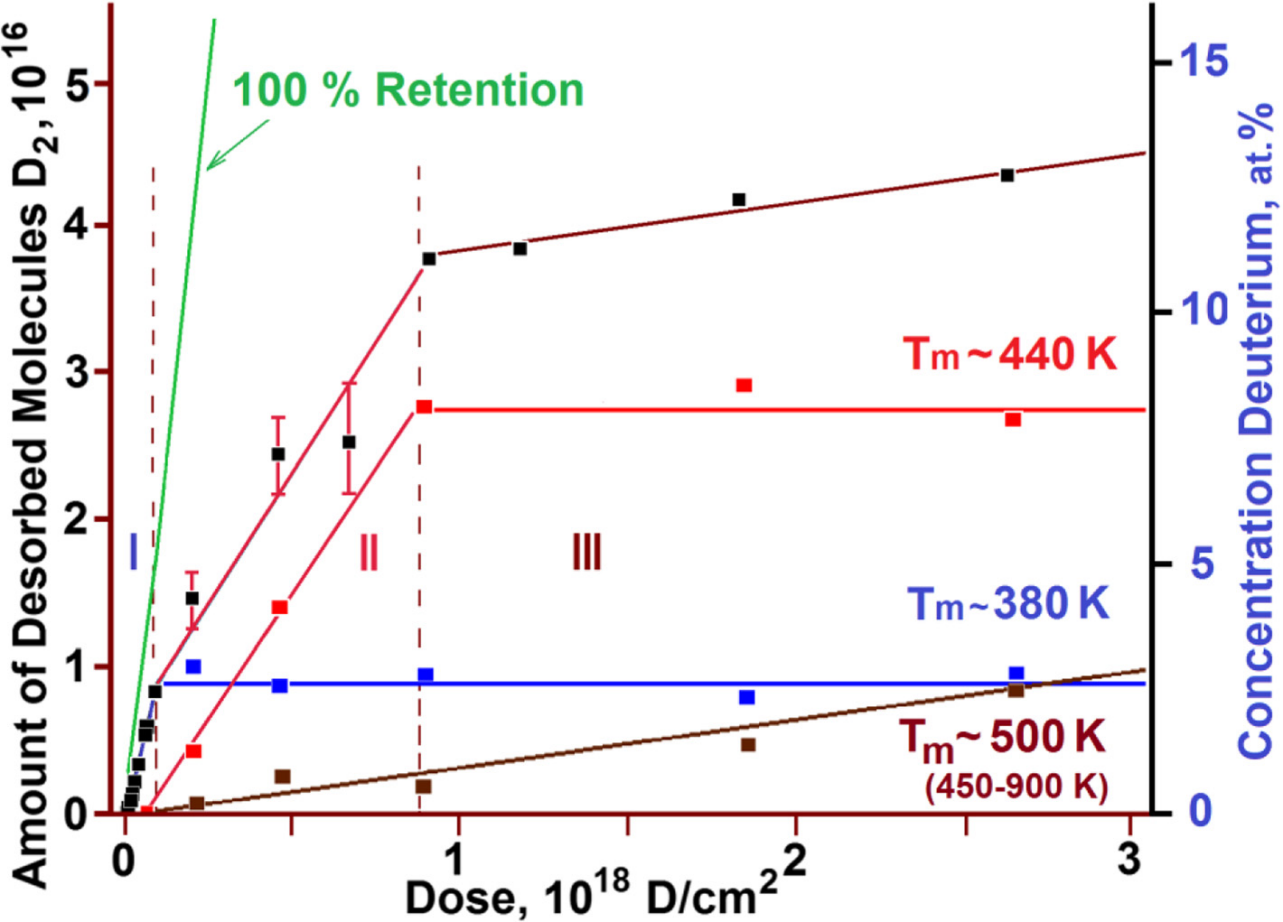
Deuterium content in each individual peak of the spectrum and the total amount of desorbed deuterium as functions of radiation dose for steel: (■)—total amount of desorbed deuterium; deuterium concentration in the peaks with maximum temperatures: (*blue square*)—*T*_m_ ~ 380 К; (*red square*)—*T*_m_ ~ 440 K; (*dark red square*)—in the 450–900 K range (*T*_m_ ~ 500 K)

Figure 10 shows the amount of deuterium in each individual peak of the TD spectrum and its total amount versus the radiation dose for austenitic steel. The curves were plotted on the basis of the data given in Figs. 7, 8, and 9. It can be seen that each kink of the curve showing the total amount of desorbed deuterium versus radiation dose correlates with variations in the behavior of the corresponding peaks in the deuterium TDS spectra. In fact, each of the peaks observed in the TDS spectra provides information on the kinetics of austenitic steel structural changes resulting from implantation of deuterium ions.

The completion of phase state formation of deuterium solid solution in steel, and correspondingly, the termination of retained deuterium concentration growth in this phase state are manifested by the decrease in the total amount of retained deuterium in steel (the peak with *T*_m_ = 380 K).

On completion of deuterium solid-solution formation in steel, the newly implanted deuterium atoms give impetus to further structural transformations, which are due to both the radiation action and the increasing concentration of implanted deuterium. In this case, the amount of retained deuterium increases at the expense of growing intensity of the high-temperature region of the spectrum with the peak temperatures 440 and 500 K. At a dose of 8 × 10^17^ D/cm^2^, the accumulation of retained deuterium at the peak with *T*_m_ = 440 K practically fully ceases, this being accompanied by the kink of the curve showing the total amount of desorbed deuterium versus the radiation dose (see Figs. 2 and 10). The deuterium concentration in this phase state remains practically unchanged during further deuterium ion implantation, and is estimated to be 7–8 at.%.

As mentioned above, beginning with the dose of 1 × 10^17^ D/cm^2^ and further, the high-temperature part of the deuterium TD spectrum increases, showing up as an extended region of deuterium desorption in the temperature range 450–900 K with *T*_m_ ~ 500 K (see Fig. 9, curve 3). The concentration growth of retained deuterium in this extended region of deuterium desorption continues up at a rate of 2 % of the implantation dose (see Fig. 9, curve 3 and Fig. 10).

### The Microstructural Change

**Fig. 11:**
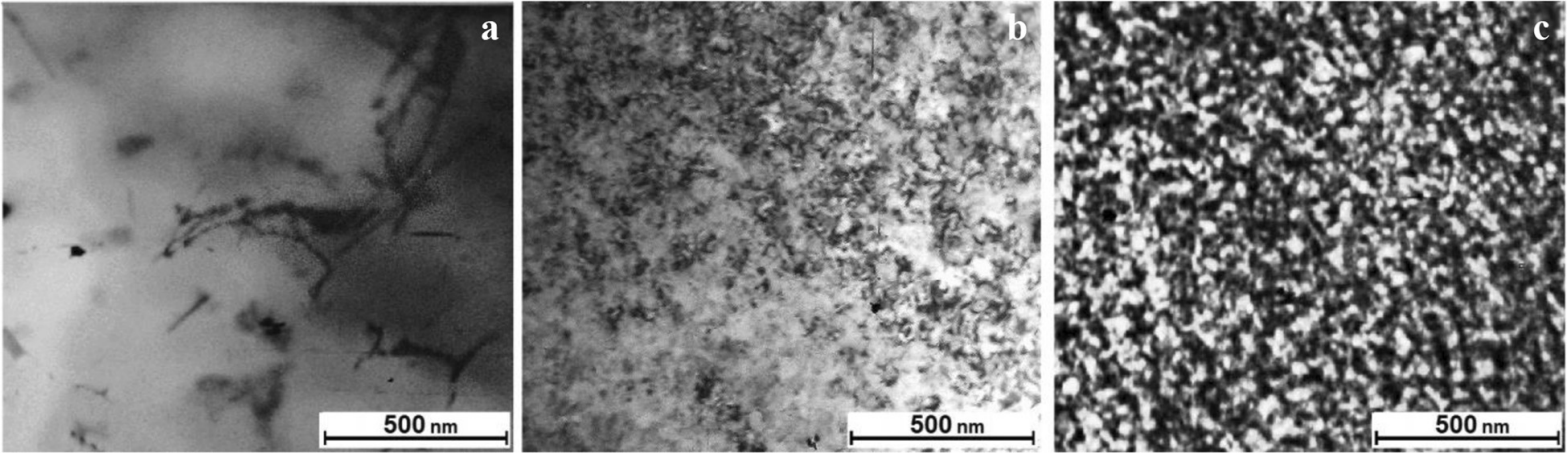
Microstructure of steel 18Cr10NiТi: **а** prior to and after irradiation with deuterium ions to the doses: **b** 5.1 × 10^17^ D/cm^2^; **с** 1.68 × 10^18^ D/cm^2^

The microstructure investigations (see Fig. 11a) have shown that the initial samples have a typical appearance of the austenitic stainless steel structure with large crystallites (pure crystal grains and crystals with inclusions randomly distributed throughout the structure).

The steel microstructure irradiated with deuterium ions to a dose of 5.1 × 10^17^ D/cm^2^ is shown in Fig. 11b. It is a characteristic of the medium-dose range (region II). It can be seen that deuterium implantation stimulates the formation of a great number of chaotically distributed dislocations, small-sized precipitates, some ordered segregations with an average scalar volume density ***ρ*** = 5 × 10^10^ cm^−2^.

The dose increase up to 1.68 × 10^18^ D/cm^2^ gives rise to the structure, which is stable against the action of deuterium ion implantation (region III). The steel microstructure exhibits the presence of a high concentration of ordered formations (nanosized crystallites) and a uniform ensemble of dislocations with the density ***ρ*** = 7 × 10^10^ cm^−2^. Actually, the basis of the formed structure is determined by the presence of nanosized crystallites and a multibranch network of intercrystalline boundaries mostly represented by the amorphous phase state (Fig. 11c). It should be stressed that as a result of radiation-induced action, in the presence of deuterium in steel, the structure has formed, which shows stability against the action of deuterium ion implantation. The presence of this structure almost entirely cancels the accumulation of newly implanted deuterium.

### Activation Energy

The thermodesorption activation energy for the peak corresponding to the solid solution of deuterium in steel was calculated using the Arrhenius equation, which determines the temperature dependence of the chemical reaction rate constant [50]:

1 $$ {\mathrm{dn}}_i/\mathrm{d}\mathrm{t}=-{K}_i\times {n}_i^{\gamma }(t)\times {e}^{-Ei/kT}, $$

where dn***/***dt is the desorption rate, which at every instant corresponds to the ordinate of the thermodesorption *i*th peak envelope;

*n*_*i*_(*t*) is the number of particles remaining in the sample by the given moment of desorption;

*K*_*i*_ is the desorption rate constant for the ***i***th peak;

*γ* is the order of reaction rate;

*E*_*i*_ is the desorption activation energy;

*k* is the Boltzmann constant;

*T* is the instantaneous temperature value.

The transformation of Eq. (1) with account of *T* = *T*_0_ + *αt* (where *α* is the rate of sample heating) and subsequent taking the logarithm lead to the following relationship

2 $$ - \ln \left[-\frac{{\mathrm{dn}}_i}{\mathrm{dT}}/{n}_i^{\gamma}\right]=-\frac{E_i}{k}\frac{1}{T}+ \ln {K}_i/\alpha . $$

**Fig. 12:**
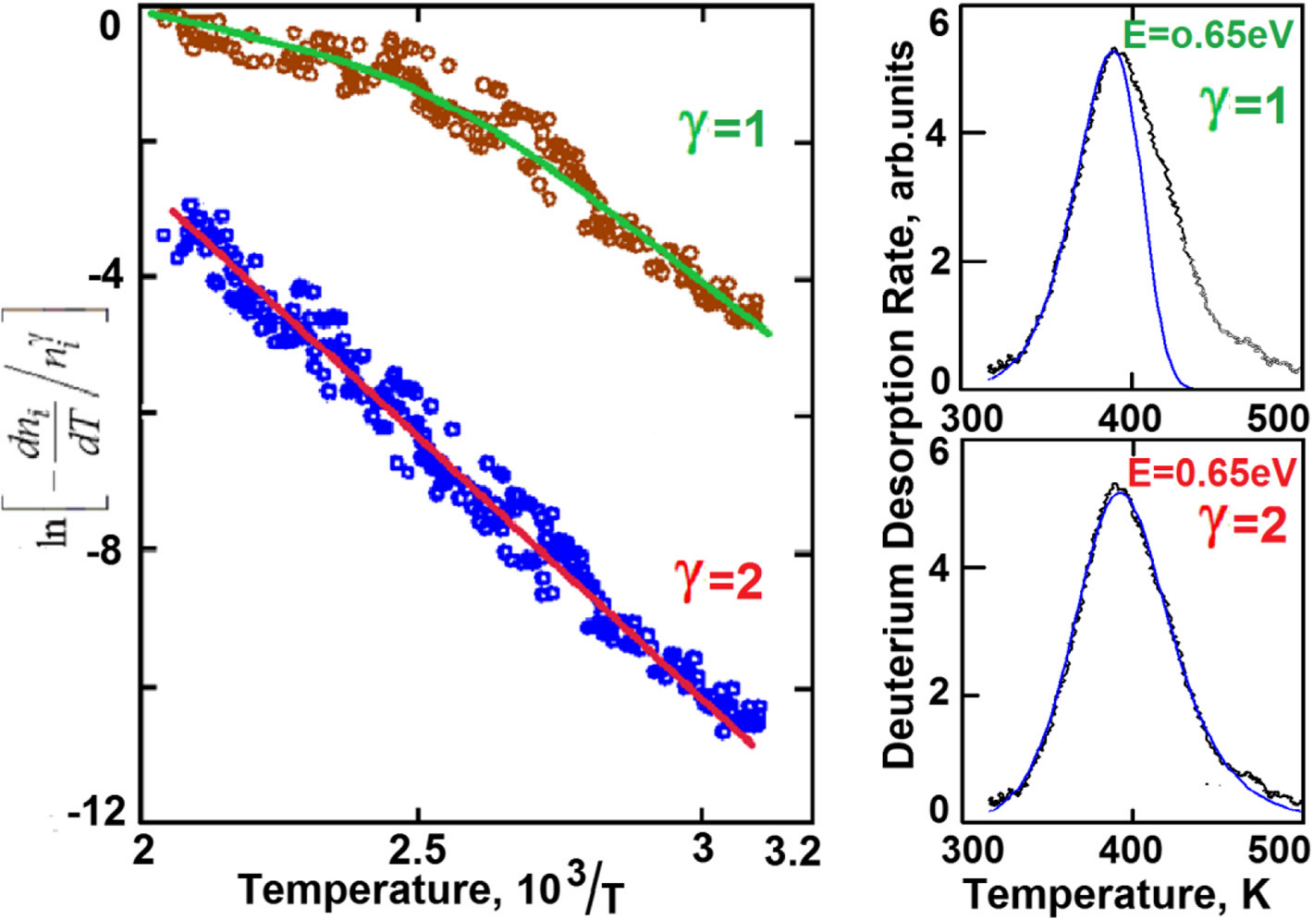
Arrhenius polyterms for the TDS spectral peak of deuterium, calculated with the first and second orders of the reaction rate, and the respective spectra with the calculated curves. The dose is 2 × 10^16^ D/cm^2^

Substituting the dn_*i*_/dT and *n*_*i*_*(T)* values from the experimentally measured spectrum of deuterium thermodesorption from steel, we have obtained the Arrhenius polyterm values for the first and second orders of the reaction rate, by which the activation energies were calculated (see Fig. 12). The activation energy for the peak with *T*_m_ = 380 K was calculated to be 0.65 eV. As is seen from the figure, the experimental points fall well on the straight line in case of the second order of the reaction, ***γ*** = 2. However, in the calculation with ***γ*** = 2, the position of the peak maximum must depend on the concentration of the components. In other words, the temperature of the maximum must shift with the radiation dose, and this is in contradiction with the obtained experimental data.

The contradiction is resolved if reasoning that in our case, we have another nature of the kinetics of solid-solution formation in the hydrogenous steel.

The Arrhenius equation calculations assume a uniform variation of the reagent concentration in the system under study, and then, with the concentration variation in the case of quadratic dependence of the desorption rate on the reagent concentration the peak temperature of the desorption must shift in the direction of decreasing temperature.

In our case, with deuterium saturation of steel, we have the formation of the solid-solution phase of deuterium in steel in the form of separate precipitates, in which the local concentration of deuterium corresponds to the ultimate stoichiometry of ~3 at.%. With an increasing concentration of deuterium in the metal, the quantity of the precipitates and their size increases until they get overlapped and the phase formation is completed. Actually, at saturation of steel with deuterium, the quantity of retained deuterium increases, while the local concentration in the precipitates remains the same. In this case, no shift of the peak temperature at deuterium desorption takes place.

### On the Nature of Retention of Deuterium Implanted in Steel at T~295 K

At room temperature, deuterium has a high diffusive mobility, the surface open to diffusion, and thereby is intensively desorbed from austenitic steel during its implantation. As is known, at room temperature, nearly all the defects formed in the course of irradiation, and above all, the Frenkel pairs are recombining [48]. However, the implanted hydrogen atoms retard the recombination of the Frenkel defects at room temperature owing to the formation of vacancy-hydrogen complexes. The interstitial metal atoms, produced in this case, take part in the formation of dislocations, crystallite interfaces, etc.

Note that the contribution of the radiation component accompanying the process of deuterium implantation was insignificant for the metals cooled down to a temperature of ~100 K, at which the diffusive mobility of deuterium is small. This distinguishing feature has made it possible to investigate the kinetics of hybrid phase formation and decay in titanium [44], palladium [43], and austenitic steel [42].

We now describe the physical processes that accompany the accumulation and retention of ion-implanted deuterium in austenitic steel being at 295 K.

At low implantation doses, deuterium is retained by stable structural formations, which are present in steel and, beside that, are formed in the process of deuterium implantation. In this case, deuterium-vacancy complexes are formed, which are randomly distributed in the implantation layer.

A further increase in the implantation dose is characterized by the process of radiation-induced action on the steel in the presence of deuterium. As a result of this action, an energy-stable steel structure is formed, which comprises nanosized steel crystallites and a multibranch network of intercrystalline boundaries. The basis for this multibranch network is provided by the amorphous state, which shows up in the TD spectra as a temperature-scale-wide region of deuterium desorption (structural formation with a varying activation energy).

The formed structure shows stability against the action of deuterium ion implantation. This manifests itself in a practically full cessation of deuterium accumulation from newly implanted ions (radiation-resistant structure). The increase in the amount of retained deuterium in the steel in the range of high implantation doses is caused by deuterium diffusion beyond the implantation volume.

## Conclusions

During the process of deuterium ion irradiation, the coefficient of deuterium retention in steel varies in discrete steps. Each of the discrete regions of deuterium retention coefficient variation in steel corresponds to different states of steel, formed during deuterium ion implantation.

Three characteristic regions with different low rates of deuterium amount desorption as the implantation dose increases, were revealed:

- I—the linear region of low implantation doses (up to 1 × 10^17^ D/cm^2^);
- II— the nonlinear region of medium implantation doses (1 × 10^17^ to 8 × 10^17^ D/cm^2^);
- III—the linear region of high implantation doses (8 × 10^17^ to 2.7 × 10^18^ D/cm^2^).

The low-dose region is characterized by deuterium retention due to stable structural formations, and also, by the formation of deuterium solid-solution phase in the steel. The total concentration of the accumulated deuterium in this region varies between 2.5 and 3 at.%.

The medium-dose region is characterized by radiation-induced action on the steel in the presence of hydrogen. The process results in the formation of the energy-stable crystalline nanostructure of steel, having a developed network of intercrystalline boundaries. The basis for this developed network of intercrystalline boundaries is provided by the amorphous state. The total concentration of the accumulated deuterium in the region of medium implantation doses makes 7 to 8 at.%.

The resulting structure shows stability against the action of deuterium ion implantation. This manifests itself in a nearly complete ceasing of deuterium accumulation from a newly implanted deuterium (radiation-resistant structure). The concentration growth of retained deuterium at a rate of ~2 % of the implantation dose continues up to the maximum dose studied here (2.7 × 10^18^ D/cm^2^). The increase in the amount of retained deuterium in the steel in the high-dose region is caused by deuterium diffusion beyond the implantation volume.

## References

[1] Alefeld G, Völkl J (1978). Hydrogen in metals I.

[2] Alefeld G, Völkl J (1978). Hydrogen in metals II.

[3] Wipf H (1997). Hydroqen in metals III. Properties and applications.

[4] Gel’d PV, Ryabov RA, Kodes ES (1979). Hydrogen and metal structure imperfections (Russian).

[5] Ageyev VN, Bekman IN, Gol’tsov VA (1987). Hydrogen interaction with metals (in Russian).

[6] Lewis FA, Aladjem A (2000). Hydrogen in metal systems. II.

[7] Varin RA, Czujko T, Wronski ZS (2009). Nanomaterials for solid state hydrogen storage.

[8] Zuttel A, Borgschulte A, Schlapbach L (2008). Hydrogen as a future energy carrier.

[9] Broom DP (2011). Hydrogen storage materials. The characterization of their storage properties.

[10] Kolachev BA (1985). Hydrogen embrittlement of metals (in Russian).

[11] Gol’tsov VA (Ed). Progress in hydrogen treatment of materials. Ukraine: Kassiopeya Ltd., Coral Gables, Donetsk; 2001. p. 544.

[12] Borchers C, Michler T, Pundt A (2008). Effect of hydrogen on the mechanical properties of stainless steels. Adv Eng Mater.

[13] Touge M, Miki T, Ikeya M (1983). Effects of X-ray irradiation on hydrogen-induced phase transformations in stainless steel. Metall Trans.

[14] Neklyudov IM, Ozhigov LS, Shilyayev BA, Laptev IN, Parkhomenko АА, Morozov AN, Bryk VV, Borodin OV (2003). Hydrogen in stainless steel in-vessel components of the WWER-1000 reactor (in Russian). Voprosy At Nauki I Tekhniki Series: Fizika radiats povr i radiats materialoved.

[15] Myers SM, Baskes MI, Birnbaum HK, Corbett JW, DeLeo GG, Estreicher SK, Haller EE, Jena P, Johnson NM, Kirchheim R, Pearton SJ, Stavola MJ (1992). Hydrogen interactions with defects in crystalline solids. Rev Modern Phys.

[16] Gavriljuk VG (2006). Austenite and martensite in nitrogen-, carbon- and hydrogen-containing iron alloys: similarities and difference. Mater Sci Eng A.

[17] Vehoff H, Wipf H (1997). Hydrogen related material problems. Hydrogen in metals III.

[18] Čížek J, Procházka I, Becvár F, Kužel R, Cieslar M, Brauer G (2004). Hydrogen-induced defects in bulk niobium. Phys Rev.

[19] Yoshida N, Ashizuka N, Fujiwara T, Kurita T, Muroga T (1988). Radiation damage and deuterium trapping in deuterium ion irradiated austenitic stainless steel. J Nucl Mater.

[20] Yoshida N, Kurita T, Fujiwara T, Muroga T (1989). Trapping of deuterium injected in austenitic stainless steel at elevated temperatures. J Nucl Mater.

[21] Čížek J, Procházka I, Brauer G, Anwand W, Mücklich A, Kirchheim R (2006). Defect studies of hydrogen-loaded thin Nb films. Appl Surf Sci.

[22] Gussev MN, Busby JT, Tan L, Garner FA (2014). Magnetic phase formation in irradiated austenitic alloys. J Nucl Mater.

[23] Shirbanda Z, Shishesaza MR, Ashrafib A (2011). Hydrogen degradation of steels and its related parameters, a review. Phase Transit.

[24] Bryk VV, Neklyudov IM (2001). Regularities of dislocation structure evolution in self-organizing materials (in Russian). Voprosy At Nauki I Tekhniki Series: Fizika radiats povr i radiats materialoved.

[25] Rudenko AG, Shilyaev BA, Voyevodin VN, Ozhigov LS (2008). Evolution of the radiation damage materials of the reactor WWER-1000 (in Russian). Voprosy At Nauki I Tekhniki Series: Fizika radiats povr i radiats materialoved.

[26] Neklyudov IM, Morozov AN, Zhurba VI, Kulish VG, Galitsky AG (2008). Hydrogen isotope retention in 18Cr10NiТi steel implanted with helium ions (in Russian). Voprosy At Nauki I Tekhniki Series: Termoyadernyi sintez.

[27] Chernov IP, Martynenko YV, Cherdantsev YP (2008). Mutual influence of hydrogen and helium in structural materials (in Russian). Voprosy At Nauki I Tekhniki Series: Termoyadernyi sintez.

[28] Neklyudov I, Morozov O, Kulish V, Azhazha V, Lavrinenko S, Zhurba V (2011). The effects of helium on temperature ranges of hydrogen isotopes retention in Hastelloy-N alloy. J Nucl Mater.

[29] Neklyudov IM, Morozov AN, Kulish VG, Zhurba VI, Galytsky AG, Piatenko EV (2009). The influence of interstitial impurities on temperature ranges of deuterium retention in austenitic stainless steel. J Nucl Mater.

[30] Murakami Y, Kanezaki T, Mine Y, Matsuoka S (2008). Hydrogen embrittlement mechanism in fatigue of austenitic stainless steels. Met Mat Trans.

[31] Teus SM, Shyvanyuk VN, Gavriljuk VG (2008). Hydrogen-induced ***γ*** 
*→* 
***ε*** transformation and the role of *ε*-martensite in hydrogen embrittlement of austenitic steels. Mater Sci Eng.

[32] Kishi A, Takano N (2010). Effect of hydrogen cathodic charging on fatigue fracture of type 310S stainless steel. J Phys Conf Ser.

[33] Mine Y, Horita Z, Murakami Y (2009). Effect of hydrogen on martensite formation in austenitic stainless steels in high-pressure torsion. Acta Mater.

[34] Rozenak P, Eliezer D (1988). Nature of the ***γ*** and ***γ******** phases in ustenitic stainless steels cathodically charged with hydrogen. Metal Trans.

[35] Rozenak P, Bergman R (2006). X-ray phase analysis of martensitic transformations in austenitic stainless steels electrochemically charged with hydrogen. Mater Sci Eng.

[36] Vakhney AG, Yaresko AN, Antonov VN, Nemoshkalenko VV (2001). The effect of hydrogen on the electronic structure and cohesive properties of iron-based alloys doped by chromium and nickel. Int J Hydrogen Energy.

[37] Tsay LW, Liu YC, Young MC, Lin D-Y (2004). Fatigue crack growth of AISI 304 stainless steel welds in air and hydrogen. Mater Sci Eng.

[38] Tsay LW, Yu SC, Huang RT (2007). Effect of austenite instability on the hydrogen-enhanced crack growth of austenitic stainless steels. Corros Sci.

[39] Gey N, Petit B, Humbert M (2005). Electron backscattered diffraction study of ε/α′ martensitic variants induced by plastic deformation in 304 stainless steel. Metal Mater Trans.

[40] Wilson KL, Baskes MI (1978). Thermal desorption of deuterium implanted stainless steel. J Nucl Mater.

[41] Pontau AE, Baskes MI, Wilson KL, Haggmark LG, Bohdansky J, Scherzer BMU, Roth J (1982). Deuterium retention in helium-damaged stainless steel: detrapping energy. J Nucl Mater.

[42] Morozov O, Zhurba V, Neklyudov I, Mats O, Rud A, Chernyak N, Progolaieva V (2015). Structural transformations in austenitic stainless steel induced by deuterium implantation: irradiation at 100 K. Nanoscale Res Lett.

[43] Rybalko VF, Morozov AN, Neklyudov IM, Kulish VG (2001). Observation of new phases in Pd-D systems. Phys Lett.

[44] Neklyudov IM, Morozov AN, Kulish VG (2005). Temperature ranges of hydride phase stability of the TiD system (in Russian). Materialovedeniye.

[45] Escobar DP, Depover T, Duprez L, Verbeken K, Verhaege M (2012). Combined thermal desorption spectroscopy, differential scanning calorimetry, scanning electron microscopy and X-ray diffraction study of hydrogen trapping in cold deformed TRIP steel. Acta Mater.

[46] Ruzhitsky VV, Gribanov YA, Rybalko VF, Khazan SM, Morozov AN, Martynov IS (1989). The multipurpose experimental facility “SKIF” (in Russian). Voprosy At Nauki I Tekhniki Series: Fizika radiats povr i radiats materialoved.

[47] Ziegler JF, Biersack JP. SRIM –The stopping and range of ions in matter: www.srim.org.

[48] Zelensky VF, Neklyudov IM, Chernyayeva TP (1988). Radiation defects and swelling of metals (in Russian).

[49] Shivachev BL, Troev T, Yoshiie T (2002). Positron lifetime computations of defects in nickel containing hydrogen or helium. J Nucl Mater.

[50] Stiler W (1989). Arrhenius equation and non-equilibrium kinetics: 100 years Arrhenius equation.

